# Do ARISCAT scores help to predict the incidence of postoperative pulmonary complications in elderly patients after upper abdominal surgery? An observational study at a single university hospital

**DOI:** 10.1186/s13741-021-00214-3

**Published:** 2021-12-08

**Authors:** Jitsupa Nithiuthai, Arunotai Siriussawakul, Rangsinee Junkai, Nutthakorn Horugsa, Sunit Jarungjitaree, Namtip Triyasunant

**Affiliations:** 1 Department of Anesthesiology, Faculty of Medicine Siriraj Hospital, Mahidol University, Bangkok, Thailand; 2 Siriraj Integrated Perioperative Geriatric Excellent Research Center, Faculty of Medicine Siriraj Hospital, Mahidol University, Bangkok, Thailand

**Keywords:** Abdominal surgery, Ageing, ARISCAT, Elderly, Postoperative, Pulmonary complications, Thai

## Abstract

**Background:**

The incidence of postoperative pulmonary complications (PPCs) is increasing in line with the rise in the number of surgical procedures performed on geriatric patients. In this study, we determined the incidence and risk factors of PPCs in elderly Thai patients who underwent upper abdominal procedures, and we investigated whether the Assess Respiratory Risk in Surgical Patients in Catalonia (ARISCAT) score helps to predict PPCs in Thais.

**Methods:**

A retrospective study was conducted on upper abdominal surgical patients aged over 65 years who had been admitted to the surgical ward of Siriraj Hospital, Mahidol University, Thailand, between January 2016 and December 2019. Data were collected on significant PPCs using the European Perioperative Clinical Outcome definitions. To identify risk factors, evaluations were made of the relationships between the PPCs and various preoperative, intraoperative, and postoperative factors, including ARISCAT scores.

**Results:**

In all, 1100 elderly postoperative patients were analyzed. Their mean age was 73.6 years, and 48.5% were male. Nearly half of their operations were laparoscopic cholecystectomies. The incidence of PPCs was 7.7%, with the most common being pleural effusion, atelectasis, and pneumonia. The factors associated with PPCs were preoperative oxygen saturation less than 96% (OR = 2.6, 1.2–5.5), albumin level below 3.5 g/dL (OR = 1.7, 1.0–2.8), duration of surgery exceeding 3 h (OR = 2.0, 1.0–4.2), and emergency surgery (OR = 2.8, 1.4–5.8). There was a relationship between ARISCAT score and PPC incidence, with a correlation coefficient of 0.226 (*P* < 0.001). The area under the curve was 0.72 (95% CI, 0.665–0.774; *P* < 0.001).

**Conclusions:**

PPCs are common in elderly patients. They are associated with increased levels of postoperative morbidities and extended ICU and hospital stays. Using the ARISCAT score as an assessment tool facilitates the classification of Thai patients into PPC risk groups. The ARISCAT scoring system might be able to be similarly applied in other Southeast Asian countries.

## Introduction

According to the World Population Prospects 2019, the proportion of individuals aged 65 years or over in South-Eastern Asia will increase from 11% in 2019 to almost a quarter in 2050 (Desa 2019). Elderly people experience progressive degenerative changes in all of their physiological systems, and they frequently have many diseases (Colloca et al. 2010). Elderly patients might not tolerate surgery well, which can result in perioperative complications.

Postoperative pulmonary complications (PPCs) happen in every age group, with an incidence ranging from less than 1 to 23% (Miskovic and Lumb 2017). PPCs affect the elderly easily because of the many changes that occur in an aging respiratory system, including the pulmonary structure, function, and neural controls. Advancing age can also cause an increase in lung compliance and a decline in chest wall compliance. In addition, the cough reflexes of the elderly might become depressed, and their ventilatory responses to hypoxia and hypercapnia impaired (Lalley 2013). In a 2006 study of PPCs, Smetana et al. found that old age is a risk factor; this finding was later supported by many studies (Miskovic and Lumb 2017; Smetana et al. 2006; Canet et al. 2010).

PPCs are mostly temporary and tend to resolve spontaneously; however, their sequelae can affect patients for a prolonged period. PPCs in the elderly are not only associated with delayed recovery, extended durations of hospital stay, and hospital readmissions, but they also contribute negatively to overall healthcare costs, quality of life, and mortality rate (Lawson et al. 2013; Shander et al. 2011).

To date, the incidences of PPCs and their sequelae at tertiary hospitals in Thailand have not been reported. The primary aim of our study was to determine the prevalence of PPCs in elderly patients, using the European Perioperative Clinical Outcome definitions of 2015 (respiratory infection, respiratory failure, pleural effusion, atelectasis, pneumothorax, bronchospasm, aspiration pneumonitis, pneumonia, and bronchitis) (Jammer et al. 2015). Scholes et al. found that patients were 50% more likely to develop PPCs with upper gastrointestinal surgery than other surgical procedures (Scholes et al. 2009). As gastrectomy, pancreatic resection, and esophagectomy have been found to have higher mortality rates than colectomy in Australia and the USA, we decided to investigate PPCs in patients undergoing upper abdominal surgery (Warrillow et al. 2008).

Our secondary outcomes were to ascertain the risk factors associated with PPCs, and the effects of PPCs on hospital stay, ICU stay, postoperative ventilator support, and mortality.

Finally, we set out to establish whether the Assess Respiratory Risk in Surgical Patients in Catalonia (ARISCAT) score can be applied to predict PPCs in the Thai population. ARISCAT scores are derived from several variables: age, oxygen saturation, respiratory tract infection in the preceding month, anemia, abdominal or thoracic surgery, operative time, and emergency surgery (Canet et al. 2010). The scoring system was developed to predict the risk of PPCs, and it demonstrated good performance in a Western European subsample (Mazo et al. 2014).

## Methods

### Objectives

The primary outcome was the incidence of PPCs in elderly patients who underwent upper abdominal surgery. The secondary outcomes were the relationships between the PPCs and various perioperative factors, including ARISCAT scores, as well as the effects those PPCs had on hospital stays, ICU stays, postoperative ventilator support, and mortality.

### Study design and participants

**Fig. 1:**
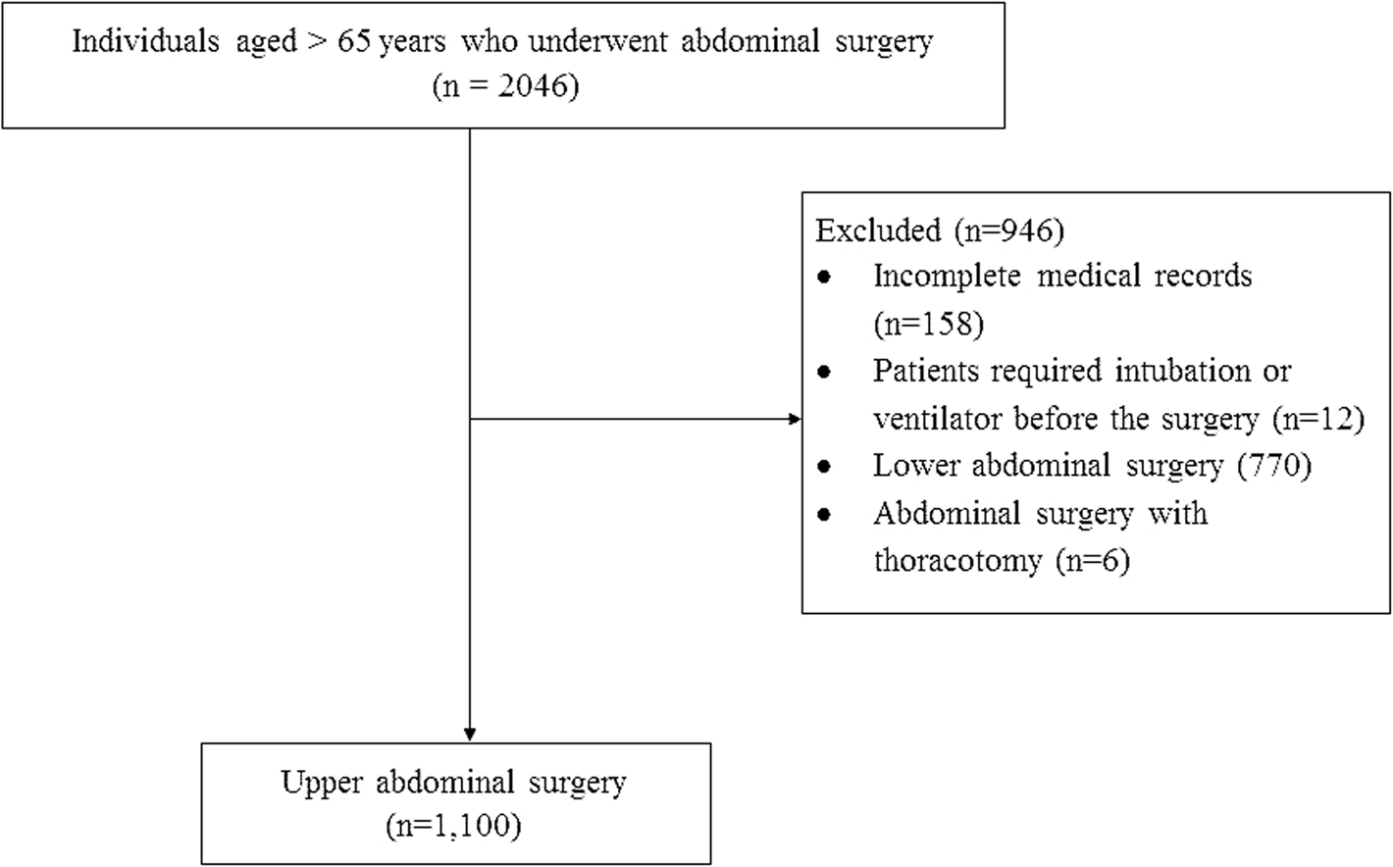
Participant recruitment

Prior approval for this retrospective study was obtained from the Siriraj Institutional Review Board (Si 006/2019). Using ICD-10-CM procedure codes, the authors searched electronic medical records to identify upper abdominal surgeries performed at Siriraj Hospital, Mahidol University, Bangkok, Thailand. The inclusion criteria were patients aged over 65 years who had undergone open or laparoscopic upper abdominal surgery. The operations included Whipple procedures, liver resections, liver transplantations, open and laparoscopic cholecystectomies, bile duct resections, pancreatectomies, adrenalectomies, splenectomies, gastrectomies, and hyperthermic intraperitoneal chemotherapies. (Fig. 1) The exclusion criteria were incomplete medical records, patients who were intubated or required a noninvasive or invasive ventilator before the surgery, thoracic surgery, and a blunt chest injury. The study period was from January 2016 to December 2019.

The sample size was calculated using the average incidence of PPCs (7.49%) obtained from a literature review (Yang et al. 2015; Jeong et al. 2014; Smith et al. 2010); the allowable error was 1.87% at a 95% level of confidence. After adding 15% to the calculated population, the study size was determined to be 900 patients. We then performed a calculation using the multiple logistic regression “rule of thumb” with 7 independent variables: smoking, oxygen saturation, recent upper respiratory infection, abnormal chest X-ray, hemoglobin, surgical duration, and urgency of surgery. With that method, the minimum number of patients required was 1100; that figure became the final sample size target.

### Assessment of postoperative pulmonary risks

Details of the cohorts’ demographic data and the factors that might be related to PPCs were retrieved from the medical records. The PPCs complied with the European Perioperative Clinical Outcome definitions of 2015 (Jammer et al. 2015).

### Statistical analysis

Comparisons were made of the characteristics of a non-PPC group and a PPC group. An analysis-of-variance model, the independent *t* test, or the Mann–Whitney *U* test was used for continuous variables; and the chi-squared or Fisher’s exact test was employed for categorical variables. Missing data were treated as missing and not imputed. Significant variables in the preliminary univariable analyses, at a predetermined alpha level of 0.2, were included in a multivariable logistic regression model. Due to the exploratory nature of our study, model building was done by analyzing the factors related to the occurrence of PPCs after surgery using stepwise backward logistic regression. Results of the multivariable logistic regression analysis were reported as adjusted odds ratio (OR) and 95% confidence interval (CI). We deemed probability (*P*) values of < 0.05 to be statistically significant. *P* values were two-sided for all statistical tests, when applicable. The data analyses were performed using IBM SPSS Statistics for Windows (version 21.0; IBM Corp., Armonk, NY, USA).

## Results

In all, 1100 elderly postoperative patients were recruited and analyzed. Their mean age was 73.6 years, and 48.5% were male. The detailed patient demographic data are listed in Table 1. There were no preoperative statistical differences in the ages, genders, body mass indexes, respiratory comorbidities, chest X-ray findings, or creatinine levels of the patients with and without PPCs.

**Table 1:** Patient demographic data

Demographic data	PPCs (***n*** = 85)	No PPCs (***n*** = 1015)	***P*** value
Age (years)	75.0 ± 7.7	73.5 ± 6.5	0.052
Male sex	46 (54.1%)	488 (48.1%)	0.285
ASA classification > 2	50 (58.8%)	452 (44.5%)	0.011
BMI (kg/m^2^)	23.7 ± 4.2	24.1 ± 4.2	0.389
Previous pulmonary disease	4 (4.7%)	31 (3.1%)	0.340
Respiratory infection in preceding month	0	7 (0.7%)	1.000
History of smoking	27 (31.8%)	229 (22.6%)	0.054
Current smoker	7 (8.2%)	38 (3.7%)	0.077
Chronic obstructive pulmonary disease	3 (3.5%)	22 (2.2%)	0.434
Asthma	2 (2.4%)	23 (2.3%)	1.000
Obstructive sleep apnea	2 (2.4%)	14 (1.4%)	0.354
Congestive heart failure	1 (1.2%)	2 (0.2%)	0.215
Preoperative SpO_2_ (%)	97.8 ± 1.5	98.3 ± 1.7	0.012
Preoperative albumin (g/dL)	3.4 ± 1.0	3.80 ± 1.2	0.002
Preoperative Hb (g/dL)	11.0 ± 2.3	11.9 ± 2.0	< 0.001
Preoperative HCO_3_	25.0 ± 3.5	25.8 ± 2.8	0.053
Preoperative creatinine	1.3 ± 1.5	1.1 ± 0.8	0.224
Abnormal CXR	24 (28.2%)	252 (24.8%)	0.486
Spirometer usage	5 (5.9%)	51 (5.0%)	0.598
Deep-breathing exercises	2 (2.4%)	23 (2.3%)	0.707

Data presented as *n* (%) or mean ± SD

*Abbreviations*: *ASA* American Society for Anesthesiologists physical status, *BMI* body mass index, *CXR* chest X-ray, *Hb* hemoglobin, *HCO*_*3*_ bicarbonate, *SpO*_*2*_ peripheral capillary oxygen saturation/pulse oximetry

The most frequent surgical operation was cholecystectomy (630 cases; 57%), with over 80% done laparoscopically. This was followed by liver resection (183 cases; 17%) and gastrectomy (65 cases; 6%).

The overall PPC incidence was 7.7% (85/1100 patients). Of those cases, pleural effusion had the highest incidence (31/85 cases; 36%), followed by atelectasis at 28% and pneumonia at 24%. The procedure with the highest prevalence of PPCs was cholecystectomy (27/85 cases; 31.8%), followed by liver resection (22/85 cases; 25.9%) and gastrectomy (9/85 cases; 10.6%). Excluding the cholecystectomies, the PPC incidence was 11.4% (61/535 patients).

The median hospital and intensive-care-unit stays of the PPC patients were significantly higher than of those without PPCs at 16 (9, 37.5) versus 5 (3, 9) days, with *P* < 0.001; and 4 (3, 10) versus 1 (1, 4) days, with *P* = 0.032, respectively.

The non-PPC and PPC groups had median ARISCAT scores of 32 (18–41; 95% CI, 31.1–32.7) and 41 (34–52; 95% CI, 40.2–46.1), respectively, with *P* < 0.001. Each score indicated an intermediate risk in the ARISCAT system. Relative to the patients with a low ARISCAT score, those with an intermediate score range had a threefold higher risk (95% CI, 1.528–6.034) of developing PPCs. By contrast, those with a high ARISCAT score range had a 7.8-fold higher probability (95% CI, 3.89–15.69). The perioperative risk factors and outcomes are listed in Tables 2, 3**,** and 4. Our multivariable regression analysis revealed that 4 risk factors were associated with PPCs: preoperative SpO_2_ ≤ 95% (OR 2.60; 95% CI, 1.23–5.51), preoperative albumin less than 3.5 g/dL (OR 1.70; 95% CI, 1.02–2.83), surgery duration exceeding 3 h (OR 2.05; 95% CI, 1.00–4.22), and emergency surgery (OR 2.84; 95% CI, 1.39–5.83). In contrast, a high incidence of PPCs was not associated with gender; body mass index; or a history of asthma, chronic obstructive pulmonary disease, or obstructive sleep apnea. On the other hand, a low PPC incidence was not related to respiratory prehabilitation therapy, such as spirometer usage or deep-breathing exercises implemented before surgery.

**Table 2:** Intraoperative parameters

Intraoperative parameters	PPCs (***n*** = 85)	No PPCs (***n*** = 1015)	***P*** value
Open surgery	60 (70.6%)	450 (44.3%)	< 0.001
Emergency surgery	14 (16.5%)	52 (5.1%)	< 0.001
Duration of surgery	3.1 (1.8, 5.0)	1.8 (1.2, 3.2)	< 0.001
Anesthesia technique			0.012
GA	51 (60%)	739 (72.8%)	
Combined GA & epidural	34 (40%)	276 (27.2%)	
Crystalloid IV (mL)	1550 (1000, 2800)	700 (400, 1700)	< 0.001
Blood loss (mL)	350 (150, 1000)	50 (10. 300)	< 0.001
Inhalation			0.147
Isoflurane	4 (4.70%)	70 (6.9%)	
Sevoflurane	21 (24.7%)	335 (33%)	
Desflurane	60 (70.6%)	603 (59.4%)	
Midazolam used	20 (23.5%)	162 (16.0%)	0.071
Neuromuscular blocking			0.005
Cisatracurium	49 (57.6%)	605(59.6%)	0.724
Atracurium	22 (25.9%)	348(34.3%)	0.115
Rocuronium	14 (16.5%)	59(5.8%)	< 0.001
Ventilator setting			0.608
VCV mode	63 (74.1%	643 (63.3%)	
PCV mode	18 (21.2%)	159 (15.7%)	
TV (cc/kg)	8.9 ± 1.2	9.0 ± 1.4	0.459
PAP (cmH_2_O)	20.7 ± 5.3	21.2 ± 4.5	0.346
RR (min)	12.2 ± 2.2	12.4 ± 2.7	0.400
PEEP (cm·H_2_O)	4.8 ± 0.83	4.8 ± 0.9	0.994
ARISCAT score	41 (34–52)	32 (18–41)	< 0.001

Data presented as *n* (%), mean ± SD, or median (IQR)

*Abbreviations*: *ARISCAT* Assess Respiratory Risk in Surgical Patients in Catalonia, *GA* general anesthesia, *IV* intravenous, *PAP* peak airway pressure, *PCV* pressure-controlled ventilation, *PEEP* positive end-expiratory pressure, *RR* respiratory rate, *TV* tidal volume, *VCV* volume-controlled ventilation

**Table 3:** Postoperative parameters

Postoperative parameters	PPCs (***n*** = 85)	No PPCs (***n*** = 1015)	***P*** value
Postoperative ventilator (h)	73 (32.5, 270)	20 (16, 81.25)	0.029
Numerical pain rating score	5.9 ± 3.2	5.3 ± 3.2	0.074
Morphine dose (mg)	6.4 ± 5.6	5.4 ± 3.2	0.647
Pethidine dose (mg)	45.0 ± 7.1	42.6 ± 12.2	0.823
Fentanyl dose (mcg)	103.3 ± 52.5	91.8 ± 41.1	0.145
Length of hospital stay (days)	16 (9, 37.5)	5 (3, 9)	< 0.001
Length of ICU stay (days)	4 (3, 10)	1 (1, 4)	0.032

Data presented as mean ± SD

*Abbreviation*: *ICU* intensive care unit

**Table 4:** Results of multivariate regression analysis

Protective/risk factors	Adjusted OR	95% CI	***P*** value
ASA classification > 2	1.30	0.80–2.09	0.290
Preoperative SpO_2_ ≤ 95%	2.60	1.23–5.51	0.012
Preop. Hb < 10 mg/dL	1.24	0.69–2.21	0.479
Preoperative albumin < 3.5 g/dL	1.70	1.02–2.83	0.040
Duration of surgery (reference < 2 h)	1		
2–3 h	1.05	0.50–2.21	0.900
> 3 h	2.05	1.00–4.22	0.050
Open surgery	1.49	0.83–2.67	0.187
Emergency surgery	2.84	1.39–5.83	0.004
Crystalloid IV > 1000 mL	1.88	0.89–4.00	0.100

*Abbreviations*: *95% CI* 95% confidence interval, *ASA* American Society for Anesthesiologists physical status, *Hb* hemoglobin, *IV* intravenous, *OR* odds ratio, *SpO*_*2*_ peripheral capillary oxygen saturation/pulse oximetry

Using Pearson’s correlations analysis, a relationship was identified between the ARISCAT score and the PPC incidence, with a correlation coefficient of 0.226 (*P* < 0.001). The area under the curve was 0.72 (95% CI, 0.665–0.774; *P* < 0.001).

## Discussion

In Thailand, the incidences of PPCs and their sequelae at tertiary hospitals have not previously been reported. Identifying their incidences and the associated, modifiable risk factors in high-risk patients might increase the quality of perioperative care and decrease overall morbidity. To this end, we collected data relating to 1100 patients aged over 65 years who had either open or laparoscopic upper abdominal surgery between January 2016 and December 2019.

The average age of the participants in our study was 73.6 years, and nearly half of the cases required minor upper abdominal surgery. Laparoscopic cholecystectomies represented 47% of all operations, and the overall incidence of PPCs was 7.7%. Excluding the cholecystectomies, the PPC incidence was 11.4%. That level was comparable with the PPC incidence of 5.8% found after abdominal surgery in an analysis of the National Surgical Quality Improvement Program by (Yang et al. 2015). Although that research team focused on major abdominal surgery, 71% of the operations were colectomies, which had the lowest rate of PPCs (4.4%) of all the operations. Moreover, the mean ages of the patients in that study were lower than in ours: 66.9 years for the PPC patients and 60.4 years for the patients without PPCs. Looking at the “Prospective Evaluation of a Risk Score for Postoperative Pulmonary Complications in Europe; PERISCOPE” data, 7.9% of patients experienced PPCs. Their median ARISCAT score was 15 (9–26), which indicated that the PPCs were low risk. Furthermore, the median age of the population was 59.1 (44.9–70.9) years. Most of the operations involved a peripheral incision (72.5%), followed by upper abdominal surgery (21.4%) and intrathoracic or cardiac surgery (6.1%) (Mazo et al. 2014).

Our investigation revealed that the PPC with the highest incidence was pleural effusion (36%), which was diagnosed from postoperative chest X-ray reports. Rossi and Bromberg also reported finding a high rate of pleural effusion through ultrasound examinations during the postoperative period following elective abdominal surgery (70.3%). Most of their cases were asymptomatic and self-limiting (Rossi and Bromberg 2005). Pleural effusions might result from sodium and water retention, and they may be aggravated by the relative cardiac decompensation typically found in the elderly (Nielsen et al. 1989). Subsequent to the performance of hepatectomies for the treatment of primary liver cancer, postoperative pleural effusions were found in a quarter of such cases. Subphrenic collection and operative injuries to the liver were found to be statistically related to those pleural effusions (Chu et al. 2007).

### Preoperative parameters

Like other studies, older age was determined to be an independent risk factor for PPCs. They were found in 7.2% of patients aged 65 to 80 years (63/881 patients) and in 10% over 80 years of age (22/219 patients). Qaseem et al. reported that, compared with younger patients, the ORs of developing PPCs were 2.09 (95% CI, 1.70–2.58) for patients aged 60–69 years, and 3.04 (95% CI, 2.11–4.39) for those aged 70–79 years. Although age cannot be modified, careful perioperative management might decrease the incidence or severity of complications in older patients (Qaseem et al. 2006).

We also found that the incidence of PPCs rose in patients having an American Society of Anesthesiologists physical status > II (crude OR 1.78; 95% CI 1.14–2.79), but it declined in those with a preoperative peripheral capillary oxygen saturation value exceeding 95% in room air (crude OR 0.30; 95%CI 0.15–0.59). Our study showed that the PPC incidence was not associated with gender, body mass index, preoperative spirometer usage, or preoperative deep-breathing exercises.

Unlike the findings of Yoder et al., we found no correlation between PPCs and respiratory comorbidities (asthma, chronic obstructive pulmonary disease, and smoking history) (Yoder et al. 2011). This may be because there were only 50 cases of respiratory-related patients, all of which were medically well controlled. Moreover, some of those 50 cases had been screened and treated by specialist staff at our Siriraj Pre-Anesthetic Clinic—with several achieving optimized medical conditions—at least 2 weeks prior to their surgeries. Although past and current cigarette smoking were not statistically associated with PPCs in our study, Lugg et al. revealed that the current smokers had a greater incidence of PPCs and a higher rate of ICU admission following non-small cell lung cancer surgery. In addition, the PPC risk was reduced following smoking cessation (Lugg et al. 2017). For various surgeries, such as spine surgery, plastic surgery, and renal transplant surgery, quitting smoking improved a wide range of outcomes. Therefore, smoking cessation should be incorporated in the pathway before surgery for better care (Grocott et al. 2017). A retrospective review conducted at Pusan National University Yangsan Hospital, South Korea, found that the incidence of PPCs after non-cardiothoracic surgery with adult asthma patients was as high as 29.1%, with the most common PPCs being pneumonia (32.4%) and bronchospasm (24.3%). The significant risk factors were age, the presence of preoperative respiratory symptoms, and a low forced expiratory volume in 1 s (Lee et al. 2014).

The mean hemoglobin levels of the 2 groups in our study being similar at around 11 g/dL, the values could not demonstrate clinical importance, with an adjusted OR of 1.24 (95% CI, 0.69–2.21). In our study, patients who had a serum hemoglobin level of < 10 g/dL had a threefold greater chance of experiencing at least 1 PPC than those with higher hemoglobin levels. Even at a mild degree (hemoglobin < 13 g/dL in males and < 12 g/dL in females), preoperative anemia was reported to be independently related to a heightened risk of 30-day mortality and morbidities (cardiac, respiratory, CNS, urinary tract, wound, sepsis, and venous thromboembolism outcomes) in patients undergoing major non-cardiac surgery (Musallam et al. 2011).

Preoperative albumin was reported to correlate inversely with complications such as reintubation, pneumonia, and failure to wean from a mechanical ventilator, especially after upper abdominal surgery (Barisione et al. 1997). Compared with colonic surgery, patients undergoing esophageal or pancreatic procedures were also found to have significantly higher complication rates at any level of serum albumin < 3.25 g/dL (Kudsk et al. 2003). The relationship between serum albumin levels and mortality was demonstrated to be continuous when the levels were < 3.5 g/dL (Gibbs et al. 1999). Although there was a statistical difference in the mean serum albumin levels of the non-PPC and PPC groups in our study, neither mean was < 3.0 g/dL, which Smetana et al. identified as a PPC predictor (Smetana et al. 2006; Lawrence et al. 2006). Patients with serum albumin levels less than 3.5 g/dL had a greater risk of PPCs, with an adjusted OR of 1.7 (95% CI, 1.02–2.83).

Preoperative spirometry usage and deep-breathing exercises showed no benefits in reducing the incidence of PPCs in our study. However, we cannot comment on the impact of this finding, given that less than 5% of our population used spirometry preoperatively, and less than 2% performed deep-breathing exercises. The preoperative physiotherapist consultations also varied, depending on the judgements of the attending surgeons, the operation type, and patient comorbidities. Furthermore, the outcomes of the prehabilitation depended on the degree of patient cooperation and compliance. In a prospective multicenter randomized controlled trial conducted in Australia and New Zealand by Boden et al., a 30-min preoperative physiotherapy education and exercise training session halved the incidence of PPCs, especially hospital-acquired pneumonia after upper abdominal surgery. The absolute risk reduction was 15% (95% CI, 7–22%), with a number needed to treat of 7 (95% CI, 5–14) (Boden et al. 2018).

### Intraoperative and postoperative parameters

In the present work, strong relationships were demonstrated between PPCs and surgical duration (especially if longer than 3 h), with a twofold increase in the incidence of complications (95% CI, 1.0–4.2). Patel et al. reported that the risk of PPCs increased with every additional minute of operating time (Patel et al. 2016).

Despite finding that laparoscopic and open cholecystectomies had similar PPC risk profiles in terms of their operative durations, Owen et al. demonstrated that open surgery had at least double the risk of PPCs compared with laparoscopic surgery (Owen et al. 2013). A separate study comparing open and minimally invasive esophagectomies reported that there was a significant reduction in postoperative pneumonia when the minimal approach was employed (Biere et al. 2012).

According to a study of the factors predicting mortality in emergency abdominal surgery of the elderly, the incidence of postoperative pneumonia was 12.8%, with over half of those occurrences being caused by aspiration. Furthermore, another 4.3% of the study cohort died from pneumonia (Fukuda et al. 2012; Ferreyra et al. 2009). By comparison, our study revealed the incidence of patients with PPCs after emergency abdominal surgery as 21.2% (14/66 cases) with adjusted OR 2.84 (95% CI, 1.39–5.83). The most common PPCs were atelectasis (6 cases), followed by pneumonia (4 cases). There was no mortality.

In patients undergoing abdominal surgery, epidural analgesia reduces the risk of postoperative pneumonia while improving pulmonary function and arterial oxygenation (Pöpping et al. 2008). However, in our study, the PPC incidence was not affected by the choice of anesthesia (general, versus a combination of general and regional anesthesia).

Looking at the major surgery subgroups in Table 5, there were no significant differences in the PPC prevalences of the patients who received general anesthesia (GA) combined with epidural anesthesia, and the other anesthetic methods. Therefore, performing combined GA-epidural anesthesia neither increased nor reduced the incidence of PPCs in our population. In addition, there were no statistical differences in the postoperative pain-rating scores or 72-h opioid consumption levels of the non-PPC and PPC groups.

**Table 5:** Major and minor surgery subgroup analysis

Factors	Major surgery (***n*** = 535)			Minor surgery (***n*** = 565)		
	PPCs (***n*** = 61, 11.4%)	No PPCs (***n*** = 474, 88.6 %)	***P*** value	PPCs (***n*** = 24, 4.2%)	No PPCs (***n*** = 541, 95.8%)	***P*** value
Duration of surgery			0.034			0.018
< 2 h	10 (7.9%)	116 (92.1%)		14 (3.1%)	432 (96.9%)	
2–3 h	9 (7.3%)	115 (92.7%)		7 (7.4%)	88 (92.6%)	
> 3 h	42 (14.7%)	243 (85.3%)		3 (12.5%)	21 (87.5%)	
Neuromuscular blocking			0.005			0.059
Rocuronium	10 (27.0%)	27 (73.0%)		4 (11.1%)	32 (88.9%)	
Others	51 (10.2%)	447 (89.8%)		20 (3.8%)	509 (96.2%)	
Combined GA with epidural anesthesia			0.838			0.459
Yes	33 (11.1%)	263 (88.9%)		1 (7.1%)	13 (92.9 %)	
No	28 (11.7%)	211 (88.3%)		23 (4.2%)	528 (95.8%)	
Maximal numerical pain score in 72 h	6.0 ± 3.2	5.4 ± 3.2	0.205	5.8 ± 3.2	5.1 ± 3.2	0.331
ARISCAT scores			0.003			< 0.001
< 26	4 (4.3%)	89 (95.7%)		3 (1.1%)	282 (98.9%)	
26–44	29 (10.2%)	256 (89.8%)		12 (5.3%)	216 (94.7%)	
≥ 45	28 (17.8%)	129 (82.2%)		9 (17.3%)	43 (82.7%)	
Total crystalloid received (mL)	2200 (1300, 3875)	1750 (1100, 2600)	0.009	950 (525, 1300)	450 (300, 650)	< 0.001
Estimated blood loss (mL)	500 (200, 1300)	300 (100, 600)	< 0.001	150 (40, 275)	10 (5, 30)	< 0.001

Data presented as mean ± SD, median (IQR)

*Abbreviation*: *ARISCAT* Assess Respiratory Risk in Surgical Patients in Catalonia

Compared with conventional ventilation, a protective ventilation strategy reduces inflammation and improves oxygenation in patients undergoing esophagectomies. In a protective ventilation group, the incidence of pneumonia was demonstrated by one study to be lower than that for a conventional ventilation group, although the difference was nonsignificant (Michelet et al. 2006). In our investigation, most of the ventilation parameters followed the lung-protective strategy; the mean tidal volumes of the PPC and non-PPC groups were almost 9 mL/kg, and the parameters of the groups were not statistically different. As to the anesthetic risk factors, no relationship was apparent between the PPCs and airway equipment, inhalation agent, or anesthetic technique.

We found an association between rocuronium and postoperative complications. The PPC-inducing action of rocuronium still remained even when used in conjunction with neostigmine or sugammadex. In a comparison of the major and minor operations, a significant difference in their PPC incidences was only demonstrated with the use of rocuronium with the major operations. In other words, the type of surgery influenced the usage of rocuronium, which then affected the occurrence of PPCs. The observational study entitled “Post-Anaesthesia Pulmonary Complications After Use of Muscle Relaxants” showed that the administration of neuromuscular blocking drugs during GA was associated with an elevated risk of PPCs (OR 1.86; 95% CI, 1.53–2.26). Furthermore, the usage of neuromuscular monitoring and reversal agents (sugammadex or neostigmine) was not associated with a decreased PPC risk (Kirmeier et al. 2019). However, in a multicenter, matched cohort analysis (STRONGER), sugammadex administration was associated with a reduction in PPC risk (adjusted OR 0.70; 95% CI, 0.63–0.77) as well as a 55% reduced risk of respiratory failure, compared with the administration of neostigmine (Kheterpal et al. 2020). In addition, a randomized controlled trial by Togioka et al. explored the effects of reversal agents on the PPC incidence of older adults undergoing prolonged surgery. Their work confirmed that sugammadex is superior to neostigmine in reducing the incidence of residual neuromuscular paralysis. Moreover, there was a threefold increase in the 30-day hospital readmission rate of the neostigmine group (15%) relative to that of the sugammadex group (5%) (Togioka et al. 2020).

A 2020 systematic review and meta-analysis focusing on the prevention of PPCs identified some perioperative interventions that probably reduce the occurrence of PPCs. For example, the use of enhanced recovery after surgery pathways and goal-directed hemodynamic therapy demonstrated benefits, whereas restrictive fluid administration strategies might not (Odor et al. 2020). Goal-directed hemodynamic therapy was a type of perioperative fluid administration that used biological targets as guidance (for example, calculated oxygen delivery, pulmonary capillary wedge pressure, or cardiac stroke volume variation). Over recent years, our institution has progressively revised the perioperative management protocol for enhanced recovery (the Siriraj ERAS Protocol). In the current work, even though we could retrospectively collect and analyze the intraoperative fluid administration for each case, we were unable to identify which fluid strategy was used. Our results showed that the median intraoperative crystalloid infusions of the PPC and non-PPC groups were 1550 mL (1000, 2800) and 700 mL (400, 1700), respectively. In the case of the major operation subgroup, the median intravenous fluid volume was 2200 mL (1300, 3875) for the PPC group, and 1750 mL (1100, 2600) for the non-PPC group (*P* = 0.009). A large intraoperative crystalloid administration was associated with a high PPC risk (adjusted OR 1.88; 95% CI, 0.89–4.00), but this might not represent statistical significance. A fluid volume cutoff point affecting PPCs could not be determined. Many factors were involved in fluid administration, like blood loss, the presence of patient comorbidities, and preoperative volume status.

An increased fluid volume administration was associated with an elevated risk of pulmonary complications, whereas goal-directed fluid administration was reported to offer a 30% reduction in pulmonary complications following upper abdominal and major vascular surgery (OR 0.7; 95% CI, 0.6–0.9) (Casado et al. 2010; Corcoran et al. 2012). During major abdominal surgery, perioperative fluid therapy recommendations aim for a moderately liberal intravenous (IV) fluid regimen, with an average crystalloid fluid infusion rate of 10–12 mL/kg/h. In addition, for higher-risk patients undergoing major surgery, employing an advanced hemodynamic monitor to assess fluid responsiveness was recommended (Miller and Myles 2019).

In our study, the median blood loss for the PPC group (350 mL [150, 1000]) was significantly higher than that for the non-PPC group (50 mL [10, 300]). This result corresponds with the finding of Sah et al. for gastric cancer treatments: blood loss volumes exceeding 500 mL were associated with early postoperative complications (OR 2.86; 95% CI, 1.67–4.92) (Sah et al. 2009). Table 5 lists the results after defining all cholecystectomies as minor surgeries. Patients who developed PPCs had considerably higher volumes of fluid administered and greater estimated blood losses. Total intravenous fluid and estimated blood loss had a linear correlation of around 82%. However, the adjusted OR of blood loss in our study was weak and nonsignificant.

Although the presence of a nasogastric (NG) tube was reported by some studies to be a PPC risk factor (Gupta et al. 2020), there were no significant differences between its use and nonuse in terms of the PPC incidences in our study. One explanation is that NG decompression was not a routine procedure at our hospital during the study period. A systematic review of prophylactic NG decompression after abdominal operations by Nelson et al. reported that patients with selective NG tube usage after laparotomies developed pneumonia and atelectasis less often than patients using NG tubes until gastrointestinal motility returned (Nelson et al. 2005). With the presence of an NG tube, patients might not cough effectively, resulting in secretion retention and atelectasis. Furthermore, the tube can trigger silent aspiration and pneumonia as the lower esophageal sphincter cannot work as it should (Mitchell et al. 1998).

The lengths of hospital and ICU stays were longer for PPC patients (Table 3). This would inevitably lead to higher costs for the patients, their families, and the healthcare system. A multicenter study concluded that even mild PPC cases—such as atelectasis and the need for prolonged oxygen therapy—were related to increases in early postoperative mortality and extended ICU and hospital stays. The researchers opined that all such cases deserve attention and intervention (Fernandez-Bustamante et al. 2017). In our study, no deaths during the perioperative period were directly attributable to PPCs.

Patients might avoid moving or breathing deeply when they feel pain or are uncomfortable, thereby possibly triggering PPCs. However, the average postoperative pain rating scores for the PPC and non-PPC groups did not differ significantly. In our analysis of the maximum postoperative pain rating scores during the first 72 post-surgery hours, the PPC patients scored a mean of 5.9 versus 5.3 for those without PPCs. Furthermore, the total opioid usage (morphine, pethidine, and fentanyl) of the 2 groups were not statistically different. However, our findings contrast with those of Roberta et al., who investigated PPCs experienced by the elderly after abdominal surgery. Those researchers concluded that pain contributes to the development of PPCs. This dissimilarity might result from the different assessment durations that were utilized. Specifically, the current investigation assessed the pain levels of both groups within 3 days postoperatively. By comparison, Roberta et al. assessed pain daily between postoperative Days 1 and 6, inclusive, and they found that their PPC patients experienced significant pain at rest on postoperative Day 4 (Shea et al. 2002).

There are many predictive tools for PPCs, for example, the ARISCAT scoring system, the LAS VEGAS risk score, the Melbourne Risk Prediction Tool, and the Surgical Lung Injury Prediction model. However, only the ARISCAT system has demonstrated sufficient predictive power in external validations (Nijbroek et al. 2019). In Thailand, only 1 risk scoring system has been validated for the prediction of respiratory complications after thoracic surgery in the Thai population (Pipanmekaporn et al. 2019). For our elderly patients undergoing upper abdominal surgery, we used the ARISCAT scoring system as a tool to predict PPCs. The median ARISCAT scores for the non-PPC and PPC groups were 32 (18–41) and 41 (34–52), respectively (*P* < 0.001). The proportions of PPCs with low, intermediate, and high ARISCAT scores were 1.9%, 8.0%, and 17.7%, respectively. Patients whose scores ranked in the intermediate-risk level had an OR for pulmonary complications of 3.04 (95% CI, 1.53–6.03), while for those with scores in the high-risk level, the OR was as high as 7.82 (95% CI, 3.90–15.70).

Table 5 showed that having an intermediate-scoring ARISCAT in the major surgery subgroup was associated with PPCs occurring twice as frequently as in the minor surgery subgroup (10.2% vs. 5.3%). In the high-scoring ARISCAT, however, there was no significant difference in the prevalence of PPCs (17.8% versus 17.3%).

A Pearson’s correlations analysis established a relationship between the ARISCAT scores and PPC incidence, with a correlation coefficient of 0.226 (*P* < 0.001). Overall, test accuracy was fair, with a value for the area under the curve of 0.72 (95% CI, 0.665–0.774; *P* < 0.001). Using a cutoff point at score 26 or analyzing the low-risk versus the intermediate-to-high-risk group, the ARISCAT score had a sensitivity of 91.8% (95% CI, 83.8–96.6%) and specificity of 36.6% (95% CI, 33.6–39.6%). The negative predictive value was as high as 98.2% (95% CI, 96.3–99.1%). At the cutoff score of 40, the sensitivity was 68.2%, while the specificity was 64.3%.

As only 85 cases out of the 1100 patients in our study cohort developed PPCs, we could not establish a predictive score that would demonstrate an effective power as well as the ARISCAT scores presently do. The ARISCAT scores did well as a screening tool for PPCs in our patients. Those scores could therefore be applied to the general Thai population and might be used in other Southeast Asian countries.

### Limitations

As this study involved a retrospective data collection, some factors that might have affected the results could not be controlled. In addition, we used the 2015 European Perioperative Clinical Outcome PPC definitions rather than those of the 2018 Standardized Endpoints for Perioperative Medicine: Core Outcome Measures in Perioperative and Anaesthetic Care. This is because the former set of definitions covers all complications predicted by the ARISCAT scoring system, whereas the latter definitions only consider four main complications (Abbott et al. 2018).

## Conclusions

PPCs are common in elderly Thai patients, being found in 7.7% of the study cohort. The factors affecting PPC incidence that have acceptable adjusted ORs are preoperative oxygen saturation, albumin level, duration of surgery, and emergency surgery. PPCs elevate the incidence of postoperative morbidity of patients and extend their lengths of ICU and hospital stays. It is appropriate to use the ARISCAT scoring system as a screening tool to classify geriatric Thai patients into PPC risk groups. The system might be able to be similarly applied in other Southeast Asian countries.

## Further research

Functional dependence status, frailty, and sarcopenia are interesting factors which might be usefully included in a further prospective study to improve PPC management.

## Data Availability

The data supporting the findings of this study are available from the Faculty of Medicine Siriraj Hospital, Mahidol University. However, restrictions apply to their availability: they were used under license for the current study and are not publicly available. Data are available from the authors upon reasonable request and with the permission of the Faculty of Medicine Siriraj Hospital, Mahidol University.

## Abbreviations

ARISCAT: Assess Respiratory Risk in Surgical Patients in Catalonia
ASA: American Society for Anesthesiologists physical status
BMI: Body mass index
CI: Confidence interval
CXR: Chest X-ray
GA: General anesthesia
Hb: Hemoglobin
HCO_3_: Bicarbonate
ICU: Intensive care unit
IV: Intravenous
NG: Nasogastric
OR: Odds ratio
PAP: Peak airway pressure
PCV: Pressure-controlled ventilation
PEEP: Positive end-expiratory pressure
PPC: Postoperative pulmonary complication
RR: Respiratory rate
SpO_2_: Peripheral capillary oxygen saturation/pulse oximetry
TV: Tidal volume
VCV: Volume-controlled ventilation

## References

[CR1] Abbott T, Fowler A, Pelosi P, De Abreu MG, Møller A, Canet J (2018). A systematic review and consensus definitions for standardised end-points in perioperative medicine: pulmonary complications. Br J Anaesth.

[CR2] Barisione G, Rovida S, Gazzaniga G, Fontana L (1997). Upper abdominal surgery: does a lung function test exist to predict early severe postoperative respiratory complications?. Eur Respir J.

[CR3] Biere SS, van Berge Henegouwen MI, Maas KW, Bonavina L, Rosman C, Garcia JR (2012). Minimally invasive versus open oesophagectomy for patients with oesophageal cancer: a multicentre, open-label, randomised controlled trial. Lancet..

[CR4] Boden I, Skinner EH, Browning L, Reeve J, Anderson L, Hill C, Robertson IK, Story D, Denehy L (2018). Preoperative physiotherapy for the prevention of respiratory complications after upper abdominal surgery: pragmatic, double blinded, multicentre randomised controlled trial. BMJ..

[CR5] Canet J, Gallart L, Gomar C, Paluzie G, Vallès J, Castillo J, Sabaté S, Mazo V, Briones Z, Sanchis J, on behalf of the ARISCAT Group (2010). Prediction of postoperative pulmonary complications in a population-based surgical cohort. Anesthesiology..

[CR6] Casado D, López F, Marti R (2010). Perioperative fluid management and major respiratory complications in patients undergoing esophagectomy. Dis Esophagus.

[CR7] Chu K-J, Yao X-P, Fu X-H (2007). Factors related to pleural effusion following hepatectomy for primary liver cancer. Hepatobiliary Pancreat Dis Int.

[CR8] Colloca G, Santoro M, Gambassi G (2010). Age-related physiologic changes and perioperative management of elderly patients. Surg Oncol.

[CR9] Corcoran T, Rhodes JEJ, Clarke S, Myles PS, Ho KM (2012). Perioperative fluid management strategies in major surgery: a stratified meta-analysis. Anesth Analg.

[CR10] Desa U (2019). World population prospects 2019: highlights.

[CR11] Fernandez-Bustamante A, Frendl G, Sprung J, Kor DJ, Subramaniam B, Ruiz RM (2017). Postoperative pulmonary complications, early mortality, and hospital stay following noncardiothoracic surgery: a multicenter study by the perioperative research network investigators. JAMA Surg.

[CR12] Ferreyra G, Long Y, Ranieri VM (2009). Respiratory complications after major surgery. Curr Opin Crit Care.

[CR13] Fukuda N, Wada J, Niki M, Sugiyama Y, Mushiake H (2012). Factors predicting mortality in emergency abdominal surgery in the elderly. World J Emerg Surg.

[CR14] Gibbs J, Cull W, Henderson W, Daley J, Hur K, Khuri SF (1999). Preoperative serum albumin level as a predictor of operative mortality and morbidity: results from the National VA Surgical Risk Study. Arch Surg.

[CR15] Grocott MP, Plumb JO, Edwards M, Fecher-Jones I, Levett DZ (2017). Re-designing the pathway to surgery: better care and added value. Perioper Med.

[CR16] Gupta S, Fernandes RJ, Rao JS, Dhanpal R (2020). Perioperative risk factors for pulmonary complications after non-cardiac surgery. J Anaesthesiol Clin Pharmacol.

[CR17] Jammer I, Wickboldt N, Sander M, Smith A, Schultz MJ, Pelosi P, Leva B, Rhodes A, Hoeft A, Walder B, Chew MS, Pearse RM, European Society of Anaesthesiology (ESA) and the European Society of Intensive Care Medicine (ESICM), European Society of Anaesthesiology, European Society of Intensive Care Medicine (2015). Standards for definitions and use of outcome measures for clinical effectiveness research in perioperative medicine: European Perioperative Clinical Outcome (EPCO) definitions. A statement from the ESA-ESICM joint taskforce on perioperative outcome measures. Eur J Anaesthesiol.

[CR18] Jeong B-H, Shin B, Eom JS, Yoo H, Song W, Han S, Lee KJ, Jeon K, Um SW, Koh WJ, Suh GY, Chung MP, Kim H, Kwon OJ, Woo S, Park HY (2014). Development of a prediction rule for estimating postoperative pulmonary complications. PLoS One.

[CR19] Kheterpal S, Vaughn MT, Dubovoy TZ, Shah NJ, Bash LD, Colquhoun DA, Shanks AM, Mathis MR, Soto RG, Bardia A, Bartels K, McCormick PJ, Schonberger RB, Saager L (2020). Sugammadex versus neostigmine for reversal of neuromuscular blockade and postoperative pulmonary complications (STRONGER): a multicenter matched cohort analysis. Anesthesiology.

[CR20] Kirmeier E, Eriksson LI, Lewald H, Fagerlund MJ, Hoeft A, Hollmann M (2019). Post-anaesthesia pulmonary complications after use of muscle relaxants (POPULAR): a multicentre, prospective observational study. Lancet Respir Med.

[CR21] Kudsk KA, Tolley EA, DeWitt RC, Janu PG, Blackwell AP, Yeary S, King BK (2003). Preoperative albumin and surgical site identify surgical risk for major postoperative complications. JPEN J Parenter Enteral Nutr.

[CR22] Lalley PM (2013). The aging respiratory system—pulmonary structure, function and neural control. Respir Physiol Neurobiol.

[CR23] Lawrence VA, Cornell JE, Smetana GW (2006). Strategies to reduce postoperative pulmonary complications after noncardiothoracic surgery: systematic review for the American College of Physicians. Ann Intern Med.

[CR24] Lawson EH, Hall BL, Louie R, Ettner SL, Zingmond DS, Han L, Rapp M, Ko CY (2013). Association between occurrence of a postoperative complication and readmission: implications for quality improvement and cost savings. Ann Surg.

[CR25] Lee SE, Jo EJ, Park HK, Cho WH, Jeon DS, Kim YS (2014). Risk factors for postoperative pulmonary complications after noncardiothoracic surgery in adult asthma patients. J Allergy Clin Immunol.

[CR26] Lugg ST, Tikka T, Agostini PJ, Kerr A, Adams K, Kalkat MS (2017). Smoking and timing of cessation on postoperative pulmonary complications after curative-intent lung cancer surgery. J Card Surg.

[CR27] Mazo V, Sabaté S, Canet J, Gallart L, de Abreu MG, Belda J, Langeron O, Hoeft A, Pelosi P (2014). Prospective external validation of a predictive score for postoperative pulmonary complications. Anesthesiology..

[CR28] Michelet P, D’Journo XB, Roch A, Doddoli C, Marin V, Papazian L, Decamps I, Bregeon F, Thomas P, Auffray JP (2006). Protective ventilation influences systemic inflammation after esophagectomy: a randomized controlled study. Anesthesiology..

[CR29] Miller TE, Myles PS (2019). Perioperative fluid therapy for major surgery. Anesthesiology..

[CR30] Miskovic A, Lumb A (2017). Postoperative pulmonary complications. Br J Anaesth.

[CR31] Mitchell CK, Smoger SH, Pfeifer MP, Vogel RL, Pandit MK, Donnelly PJ, Garrison RN, Rothschild MA (1998). Multivariate analysis of factors associated with postoperative pulmonary complications following general elective surgery. Arch Surg.

[CR32] Musallam KM, Tamim HM, Richards T, Spahn DR, Rosendaal FR, Habbal A, Khreiss M, Dahdaleh FS, Khavandi K, Sfeir PM, Soweid A, Hoballah JJ, Taher AT, Jamali FR (2011). Preoperative anaemia and postoperative outcomes in non-cardiac surgery: a retrospective cohort study. Lancet..

[CR33] Nelson R, Tse B, Edwards S (2005). Systematic review of prophylactic nasogastric decompression after abdominal operations. Br J Surg.

[CR34] Nielsen PH, Jepsen SB, Olsen AD (1989). Postoperative pleural effusion following upper abdominal surgery. Chest..

[CR35] Nijbroek SG, Schultz MJ, Hemmes SN (2019). Prediction of postoperative pulmonary complications. Curr Opin Anaesthesiol.

[CR36] Odor PM, Bampoe S, Gilhooly D, Creagh-Brown B, Moonesinghe SR (2020). Perioperative interventions for prevention of postoperative pulmonary complications: systematic review and meta-analysis. BMJ..

[CR37] Owen RM, Perez SD, Lytle N, Patel A, Davis S, Lin E (2013). Impact of operative duration on postoperative pulmonary complications in laparoscopic versus open colectomy. Surg Endosc.

[CR38] Patel K, Hadian F, Ali A, Broadley G, Evans K, Horder C, Johnstone M, Langlands F, Matthews J, Narayan P, Rallon P, Roberts C, Shah S, Vohra R (2016). Postoperative pulmonary complications following major elective abdominal surgery: a cohort study. Perioper Med (Lond).

[CR39] Pipanmekaporn T, Bunchungmongkol N, Punjasawadwong Y, Lapisatepun W, Tantraworasin A, Saokaew S (2019). A risk score for predicting respiratory complications after thoracic surgery. Asian Cardiovasc Thorac Ann.

[CR40] Pöpping DM, Elia N, Marret E, Remy C, Tramer MR (2008). Protective effects of epidural analgesia on pulmonary complications after abdominal and thoracic surgery: a meta-analysis. Arch Surg.

[CR41] Qaseem A, Snow V, Fitterman N, Hornbake ER, Lawrence VA, Smetana GW, Weiss K, Owens DK, Aronson M, Barry P, Casey de Jr, Cross JT Jr, Fitterman N, Sherif KD, Weiss KB, Clinical Efficacy Assessment Subcommittee of the American College of Physicians (2006). Risk assessment for and strategies to reduce perioperative pulmonary complications for patients undergoing noncardiothoracic surgery: a guideline from the American College of Physicians. Ann Intern Med.

[CR42] Rossi LA, Bromberg SH (2005). Estudo prospectivo do derrame pleural pós-cirurgia abdominal e dos fatores de risco associados: avalição por ultra-sonografia. Radiol Bras.

[CR43] Sah BK, Zhu ZG, Chen MM, Xiang M, Chen J, Yan M, Lin YZ (2009). Effect of surgical work volume on postoperative complication: superiority of specialized center in gastric cancer treatment. Langenbecks Arch Surg.

[CR44] Scholes RL, Browning L, Sztendur EM, Denehy L (2009). Duration of anaesthesia, type of surgery, respiratory co-morbidity, predicted VO2max and smoking predict postoperative pulmonary complications after upper abdominal surgery: an observational study. Aust J Physiother.

[CR45] Shander A, Fleisher LA, Barie PS, Bigatello LM, Sladen RN, Watson CB (2011). Clinical and economic burden of postoperative pulmonary complications: patient safety summit on definition, risk-reducing interventions, and preventive strategies. Crit Care Med.

[CR46] Shea RA, Brooks JA, Dayhoff NE, Keck J (2002). Pain intensity and postoperative pulmonary complications among the elderly after abdominal surgery. Heart Lung.

[CR47] Smetana GW, Lawrence VA, Cornell JE (2006). Preoperative pulmonary risk stratification for noncardiothoracic surgery: systematic review for the American College of Physicians. Ann Intern Med.

[CR48] Smith PR, Baig MA, Brito V, Bader F, Bergman MI, Alfonso A (2010). Postoperative pulmonary complications after laparotomy. Respiration..

[CR49] Togioka BM, Yanez D, Aziz MF, Higgins JR, Tekkali P, Treggiari MM (2020). Randomised controlled trial of sugammadex or neostigmine for reversal of neuromuscular block on the incidence of pulmonary complications in older adults undergoing prolonged surgery. Br J Anaesth.

[CR50] Warrillow SJ, Bellomo R, Birkmeyer J, Davey P (2008). Major surgery in Victoria and the United States: a comparison of hospital mortality in older patients. Crit Care Resusc.

[CR51] Yang CK, Teng A, Lee DY, Rose K (2015). Pulmonary complications after major abdominal surgery: National Surgical Quality Improvement Program analysis. J Surg Res.

[CR52] Yoder MA, Sharma S, Hollingsworth H (2011). Perioperative pulmonary management.

